# Notes and News

**Published:** 1900-01-20

**Authors:** 


### NOTES AND NEWS.

THE Erasmus Wilson Lectures will be delivered at the Royal College of Surgeons on February 12th, 14th, and 16th, by Mr. Treacher Collins, assistant surgeon to the Royal London Ophthalmic Hospital.

THE London University will probably take up its new quarters at the Imperial Institute in May. The Government has granted the eastern and central portions of the institute for the purpose.

THE services of Professor McFadyean, of the Royal Veterinary College, have been enlisted to investigate the epidemic of pink eye which has broken out among the horses at Liverpool.

A NEW block has been opened at the City Hospital, Aberdeen. The block is devoted to administrative purposes, and the accommodation it provides was much needed. The cost is estimated at £5,000.

IT is not without interest to note that in the medical establishment of the Czar there are one chiropodist and one honorary chiropodist. Here then is an opening for another auxiliary profession, besides those of the midwives and the spectacle makers.

ADDITIONS and improvements have been recently made at the Bournemouth Royal Victoria Hospital. These form part of the Jubilee Memorial of Bournemouth. The out-patients' department has been enlarged and improved, and a fine operating theatre added.

A NEW infirmary has been opened at Liskeard Workhouse. The accommodation is for 60 patients, and the cost has been £5,500. A feature in the building is the provision of small wards to enable isolation to be carried out, and this will be done in cases of phthisis.

DAVOS is said to be having bad weather, and, indeed, to be in a "slushy" state this winter, so that people who are hoping to get benefit from the dry atmosphere, which forms an essential part of the "mountain cure," would do well to make sure of the climatic conditions prevailing at their favourite resort before starting.

IT is announced that Mr. Alfred Fripp, Surgeon-in-Ordinary to the Prince of Wales and assistant surgeon at Guy's Hospital, will be the chief surgeon in charge of the proposed Yeomanry Hospital.

IMPORTANT measures have been taken by the Indian Government to regulate the embarkation of pilgrims to Mecca. They are only to be allowed to embark at the port of Chittagong, after residence in the camp there for one year. By this measure it is hoped that the danger of spreading the plague in Europe will be minimised.

THE many deaths from accidental shooting by young people have called forth a demand that the laws restricting the sale or use of firearms amongst boys should be rendered more stringent. Parallel with this plea for grandmotherly legislation we have a complaint in the opposite direction. This is that through the present prohibitory gun licence the youth of England has little opportunity of acquiring skill in shooting, and the superior advantages of the young Boers are cited as being responsible for producing an army of skilled marksmen without any organised training.

ACCORDING to the weekly return of the Registrar-General the mortality last week fell somewhat from the high level attained the week before, namely, from 37·1 to 33·3 per 1,000 per annum, but was still 10·0 per 1,000 above the mean rate for the corresponding period. The deaths referred to diseases of the respiratory organs had also fallen from 1,221 the week before to 994 last week, but were still 260 above the corrected average. On the other hand, the deaths directly attributed to influenza continued to mount up, having been 340 last week, against 316 the week before, and only 69 three weeks before.

IN Hong Kong efforts to check the spread of the disease of a very different nature are likely to be undertaken by the recommendation of the sanitary board. They recommend a crusade against rats, now acknowledged to be amongst the chief disseminators of the disease. It is proposed to offer a reward for every rat offered, dead or alive. Of course, there is a danger in a system of reward for vermin so quickly bred as rats; but, on the other hand, some inducement is necessary to enlist the co-operation of the native population, who attach little importance to sanitary measures.

THE details of a recent small outbreak of small-pox at the Eastern Fever Hospital, which were reported to the meeting of the Metropolitan Asylums Board last Saturday were somewhat curious. It appears that on December 7th a patient was admitted to that institution certified by one of the medical staff at the German Hospital to be suffering from scarlet fever. Two days later the patient was removed to the South Wharf, where he was seen by Dr. Ricketts, medical superintendent of the hospital ships, who pronounced the case to be one of hæmorrhagic small-pox. The patient was thereupon taken to the hospital ships, where he died. On December 20th a patient suffering from scarlet fever in the Eastern Hospital was also diagnosed to be affected with small-pox, and was removed to the South Wharf; and on subsequent days other patients similarly affected were removed, making seven in all. It was stated that arrangements had been made for their reception at the Gore Farm Lower Hospital.

